# Relatively Long Survival in Hepatocellular Carcinoma Presenting With Carcinoid Syndrome

**DOI:** 10.4021/gr2010.02.171w

**Published:** 2010-01-20

**Authors:** Sylvester Chuks Nwokediuko, Ijoma Uchenna, Ofoegbu Esther, Okafor Okechukwu, Onuh Augustine, Ajuyah Charity

**Affiliations:** a Departments of Medicine, University of Nigeria Teaching Hospital Ituku/Ozall, Enugu, Nigeria; b Departments of Morbid Anatomy, University of Nigeria Teaching Hospital Ituku/Ozall, Enugu, Nigeria; c Departments of Radiation Medicine, University of Nigeria Teaching Hospital Ituku/Ozall, Enugu, Nigeria; d Divine Charity Clinic, 6 Emaya Lane, Near Peemos Place GRA, Warri, Nigeria

**Keywords:** Carcinoid syndrome, Hepatocelluar carcinoma, Paraneoplastic syndrome, Survival

## Abstract

Hepatocelluar carcinoma is one of the commonest cancers in Nigeria. Some patients may manifest a variety of paraneoplastic syndromes. Carcinoid syndrome is an extremely rare presentation of hepatocellular carcinoma. A 57-year old man presented with recurrent facial flushing and diarrhea, tricuspid regurgitation, and very high level of urinary hydroxyindoleacetic acid (HIAA) as the first manifestation of a multicentric hepatic lesion which proved histologically to be hepatocellular carcinoma. The lesions also exhibited arterial hypervascularization on contrast enhanced computerized tomography. The patient is still alive after 6 years of symptoms.

## Introduction

The carcinoid syndrome, which consists of cutaneous flushing, bronchospasm, diarrhea and right side cardiac valvular lesions, was described by Pernow and Waldenstrom in 1954 [1]. It develops when the vasoactive substances produced by a carcinoid tumor escape hepatic degradation and gain access into the systemic circulation. However, carcinoid tumors that directly release hormones into the systemic circulation, for example, bronchial carcinoids may present with carcinoid syndrome in the absence of hepatic metastasis. Conventional sites for carcinoid tumors include appendix, ileum, rectum and bronchi. Primary hepatic carcinoid tumours are extremely rare and when they occur, carcinoid syndrome is very rarely the presenting feature [2-4]. Carcinoid syndrome has also been reported with bronchial adenoma, oat cell carcinoma of the lung, medullary carcinoma of the thyroid, retroperitoneal neuroblastoma, undifferentiated carcinoma of the cervix, biliary tract tumors, ovarian teratomas, and with gastric and pancreatic cancers [5-10].

In this case report we present a 57-year old man in whom carcinoid syndrome was the first presentation of an underlying malignant disease which turned out on investigation to be hepatocellular carcinoma (HCC). He is alive and relatively stable 6 years after onset of symptoms.

## Case Report

A 57-year old Nigerian trader was first seen in the medical outpatient clinic of the University of Nigeria Teaching Hospital Ituku/Ozalla on February 8th, 2006 with complaints of recurrent facial flushing for 2 years. The flushing occured episodically at a frequency of once a day, lasting for 5 - 10 minutes. About 2 months prior to presentation, the flushing attacks became more frequent, occurring several times a day with each episode lasting for 30 minutes to 1 hour. The attacks were usually provoked by anger, addressing a crowd and sexual intercourse. A typical episode starts from the head and neck with a peppery feeling which quickly spreads to the trunk and entire body. This is quickly followed by an erythematous discoloration of the face and trunk, swelling of the face and redness of the eyes. The patient also complained of recurrent diarrhea but there was no history of cough, chest tightness or wheezing. About 4 years after the onset of flushing he developed recurrent shortness of breath on exertion and ankle swelling.

He had an illness about 20 years earlier which was characterized by jaundice. He took alcoholic drinks significantly, about 4 bottles of beer daily for over 15 years.

Physical examination revealed an apparently healthy-looking man who did not have any peripheral stigmata of liver disease. He had hepatomegaly and a pansystolic murmur at the lower sternal edge. The clinical impression made at this stage was carcinoid syndrome. The following investigation results were obtained. Abdominal ultrasonography showed multiple rounded masses in the liver which were presumed by the radiologist to be due to metastasis to the liver. Hepatitis B surface antigen (HBsAg) was positive but Hepatitis C virus (HCV) antibody was negative. Chest x-ray did not show any pulmonary abnormality. Barium enema and upper gastrointestinal endoscopy did not show any lesion. Liver function test, total serum protein, serum albumin, fasting blood sugar, serum cholesterol, serum electrolytes, urea and creatinine all showed values within the normal ranges. Full blood count and prothrombin time were normal. Antibodies to HIV 1 and 2 were negative. Prostatic specific antigen was less than 4 ng/ml (normal = 0 - 4 ng/ml), and alpha fetoprotein was less than 10 ng/ml (normal ≤ 30 ng/ml). Twenty-four hour urine hydroxy-indoleacetic acid (HIAA) was 960.1 mmol/dl (normal = 10.4 - 41.6 mmol/dl).

**Figure 1:**
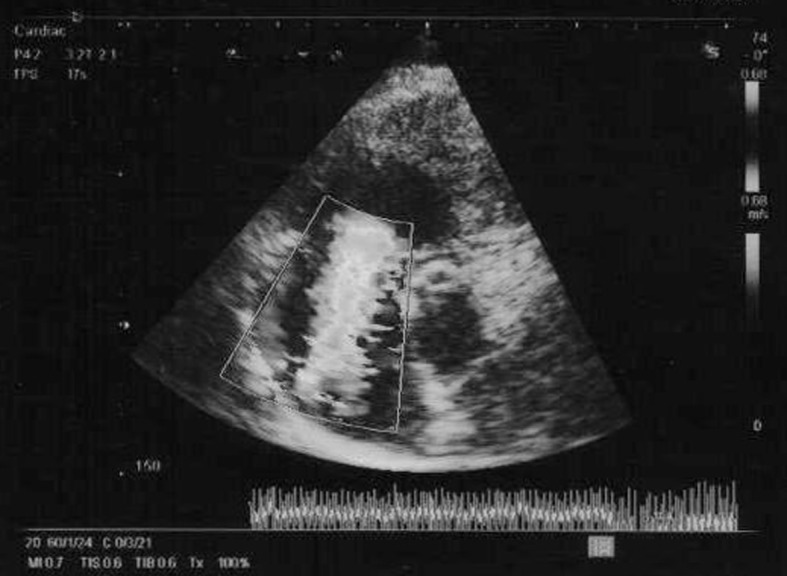
Two dimensional echocardiography showed a hyperactive, dilated right ventricle with rigid immobile tricuspid valve leaflets and moderately severe tricuspid regurgitation, dilated right atrium and normal aortic and pulmonary valves.

Two dimensional echocardiography showed a hyperactive, dilated right ventricle with rigid immobile tricuspid valve leaflets and moderately severe tricuspid regurgitation, dilated right atrium and normal aortic and pulmonary valves (Fig. 1).

Computerized axial tomography of the abdomen in spiral mode with oral and intravenous contrast showed an enlarged liver which harboured multiple hypoechoic masses of varying sizes which showed avid but patchy contrast uptake at early arterial phase involving both lobes. No signs of portal vein obstruction or thrombosis were seen. The masses were poorly defined, lacked capsules of any sort and appeared isoechoic with the liver on later scans. The bowel loops appeared normal with no signs of desmoplastic reaction or small bowel obstruction. The colon and rectum were completely distended by gas and showed no masses or areas of stricture. Detailed search of the right iliac fossa revealed no masses. The impression was that of an early arterial phase enhancing multiple hepatic masses raising a strong suspicion of multicentric hepatoma.

**Figure 2:**
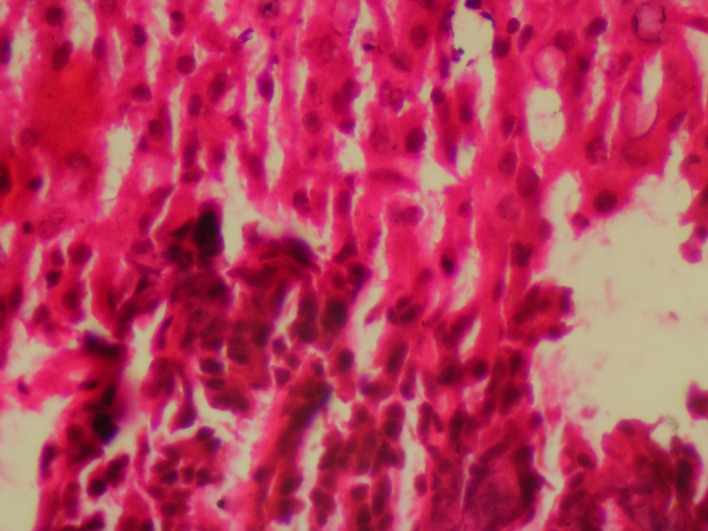
Histopathology showed atypical hepatocyte clusters with large hyperchromatic pleomorphic nuclei with scanty cytoplasm, reactive fibrosis and areas of fatty change and spotty necrosis.

Histopathology report of liver biopsy showed atypical hepatocyte clusters with large hyperchromatic pleomorphic nuclei with scanty cytoplasm, reactive fibrosis and areas of fatty change and spotty necrosis. Features were suggestive of hepatocellular carcinoma (Fig. 2).

The patient is currently on subcutaneous octreotide, with good effect. The flushing attacks and the diarrhea have reduced considerably but tend to recur whenever the patient misses the medication, usually as a result of financial constraints. The shortness of breath and ankle swelling are satisfactorily controlled with diuretics (frusemide and spironolactone).

## Discussion

Hepatocelluar carcinoma (HCC) is one of the commonest malignancies in Africa and indeed the commonest cancer in African males [11]. Most patients present at a time when curative therapy is virtually impossible. The commonest presenting features are abdominal pain, abdominal mass, easy satiety and leg swelling [12]. The prognosis is usually dismal as most of the patients die within 6 months of diagnosis [13].

The patient being reported presenting with typical carcinoid syndrome and histology of the hepatic lesion is consistent with HCC. Most patients with HCC in Nigeria rarely survive beyond 6 months from the onset of symptoms. It is interesting to note that this patient has remained alive and relatively stable for 6 years in spite of the histology of the tumor.

Primary hepatic carcinoid tumor is rare and still appears in literature as case reports. Carcinoid syndrome in primary hepatic carcinoid tumors is even rarer with only 3 reported cases [2, 10, 14]. This case is actually the 4th in literature and the first in Nigeria in spite of the high incidence and prevalence of this cancer in sub Saharan Africa.

Relatively long survival is a feature of carcinoid tumors and has been reported as better in primary hepatic carcinoids [15]. Cells undergoing neoplastic transformation may acquire new humoral synthetic pathways, especially the capacity to synthesize protein hormone. It is believed that production of hormones by non-endocrine tumours may be the result of genetic derepression during neoplastic transformation which then permits synthesis of substances not characteristic of the normal and/or differentiated cells of that organ [10]. The alternative explanation is that a mutational change in the neoplastic cell may result in acquisition of enzymes which would then complete a previously absent biochemical pathway. It does appear that when carcinoid tumors occur in non endocrine organs such as the liver, they acquire the phenotype of carcinoid tumors rather than that of the non endocrine organ and that may explain the relatively long survival. Carcinoid tumors are generally regarded as tumors of borderline malignancy. This knowledge may have therapeutic implication in the ongoing effort to discover effective therapy for cancers including HCC.

During the clinical course of HCC, patients may manifest a variety of paraneoplastic syndromes. These syndromes are symptom complexes that cannot be readily explained, either by the local or distant spread of the tumour or by the elaboration of hormones indigenous to the liver. Several studies including those conducted in Nigeria have documented erythrocytosis, hypoglycaemia, hypercalceamia, and hypercholesterolemia as the major paraneoplastic syndromes of HCC [16, 17]. Carcinoid syndrome is an extremely rare manifestation of HCC [10]. Despite the high incidence of HCC in Nigeria, there has not been any report of HCC presenting with carcinoid syndrome.

A diagnosis of primary hepatic carcinoid tumor requires the exclusion of tumor at more common primary sites. This entails extensive work up [18]. Abdominal ultrasonography was one of the first investigations carried out on this patient and the findings raised a strong suspicion of metastasis to the liver from a carcinoid tumour. A meticulous search for this primary site using computerized tomography (CT) scan failed to show any lesion in the typical locations of carcinoid tumor (appendix, ileum, rectum and bronchi). Arterial hypervascularisation was clearly demonstrated in the hepatic lesions in CT scan after contrast injection. This phenomenon is fairly specific for HCC and that explains its inclusion in the proposed non-invasive criteria for the diagnosis of HCC in cirrhotic patients [19].

The patient also has some known risk factors for liver disease. He has evidence of previous infection by Hepatitis B virus (HBV) as shown by HBsAg seropositivity. There was also a strong history of significant alcohol consumption. Histopathology of liver biopsy showed features typical of HCC of well differentiated variety. It is only when a cell is reasonably differentiated that it may be able to carry out metabolic function including synthesis and secretion of hormones. The processes leading to hormone production are too intricate for a poorly differentiated cell to accomplish.

Tumors that arise from organs that are embryologically derived from the endoderm of the primitive foregut commonly produce the carcinoid syndrome [20]. Since hepatocytes are derived from the same anlage, it is not unexpected that HCC might also produce carcinoid syndrome. However, immunohistochemical studies could not be carried out to further elucidate the cellular origin of the tumor due to lack of facilities for this modality of investigation.

Alpha fetoprotein (AFP) was not significantly elevated in the patient’s serum. This is not surprising because previous studies have found wide variations in AFP validity in the context of HCC diagnosis [21-23]. Ethnic differences are also known to affect the expression of this oncofetal protein by HCC [24, 25].
